# Effect of Partial vs Full Disclosure of Potential Assignment to Placebo on Participant Blinding, Perceptions of Group Assignment, and Trial Outcomes: A Randomized Clinical Trial

**DOI:** 10.1001/jamanetworkopen.2022.4050

**Published:** 2022-03-24

**Authors:** Jiyoon Won, Heejung Bang, Hyangsook Lee

**Affiliations:** 1 Department of Science in Korean Medicine, College of Korean Medicine, Graduate School, Kyung Hee University, Seoul; 2 Division of Biostatistics, Department of Public Health Sciences, University of California, Davis

## Abstract

This randomized clinical trial investigates the effect of partial vs full disclosure of potential assignment to placebo on participant blinding, perceptions of group assignment, and clinical outcomes.

## Introduction

Successful participant blinding in a placebo-controlled randomized clinical trial (RCT) strengthens the credibility of the results, because knowledge of the assignment and perception of the treatment may affect outcomes. During the informed consent process in RCTs, the current standard is to provide full disclosure (FD) regarding the placebo or control selected. However, in some nonpharmacologic trials, partial disclosure (PD) may be used. A review reported that this difference in disclosure (FD vs PD) might affect participant blinding, and the pooled estimate of between-group effect sizes could be larger with PD than FD.^1^ Studies that directly compare different placebo information disclosures are lacking. Therefore, we tested the effect of FD vs PD on participant blinding and clinical outcomes of acupuncture and delayed-onset muscle soreness.

## Methods

This RCT was approved by the Kyung Hee University Ethics Committee (trial protocol appears in Supplement 1). Volunteers provided written informed consent. The trial followed the Consolidated Standards of Reporting Trials (CONSORT) guideline.

The trial was conducted from October to December 2019. Participant recruitment is described in eAppendix 1 of Supplement 2; randomization is shown in the Figure. In total, 89 healthy adult volunteers were randomized to 1 of 4 groups: real acupuncture (RA) and FD, RA and PD, placebo acupuncture (PA) and FD, and PA and PD. The patient information leaflet (PIL) for the FD group stated that control acupuncture is simulated acupuncture; the PIL for the PD group stated that control acupuncture has been used in clinical trials as a comparison to experimental acupuncture. The study description provided to patients and all procedures are described in eAppendix 1 in Supplement 2.

**Figure.**
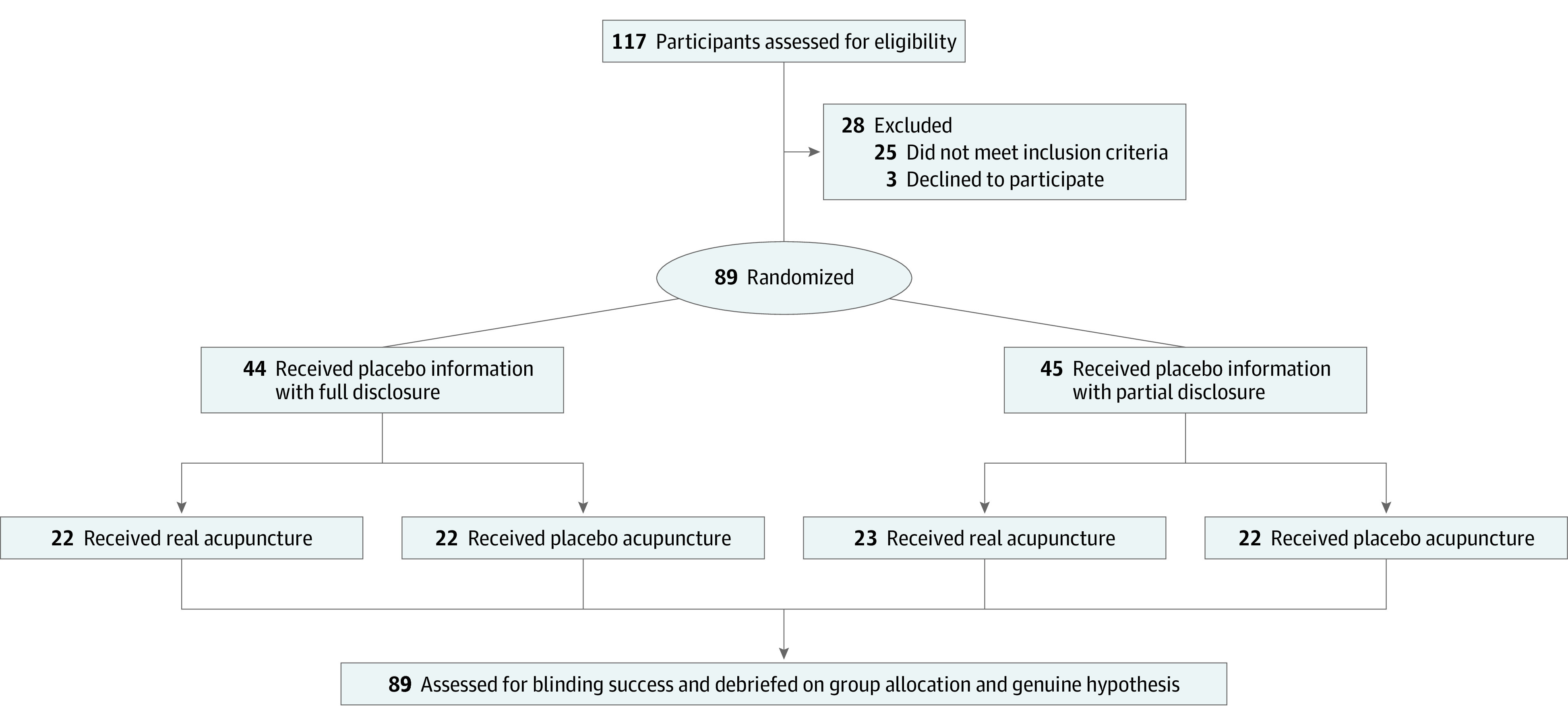
Study Flow Diagram

Delayed-onset muscle soreness was induced on the biceps brachii of the nondominant arm with eccentric exercise (eAppendix 2 in Supplement 2). The allocated acupuncture treatment was given, and pain and discomfort were assessed once daily for 3 days using an algometer and a visual analog scale. Placebo acupuncture was identical to RA except that nonpenetrating acupuncture needles replaced actual needles (eAppendix 3, eFigure in Supplement 2).^2^ On day 3, participants were debriefed about the study hypothesis and their acupuncture assignment.

The primary outcome, blinding property, was measured with a blinding index (BI), calculated as the difference in the estimated probabilities of correct vs incorrect guesses (>0 [null] indicate a more correct guess). For interpretation, the BI values were categorized as more correct (≥0.2), random (−0.2 < BI < 0.2), or incorrect (≤−0.2) and another ideal blinding (−0.2 < summed BI < 0.2).^3,4^

The BI was calculated and blinding scenarios were descriptively interpreted,^3,4^and pain and discomfort data were subjected to mixed analysis of variance with a significance level at *P* < .05 (2-sided). All statistical analyses were performed with SPSS, version 25.0 (IBM).

## Results

Of 89 participants, 40 were men and 49 were women (Figure). Participants had a mean (SD) age of 26.6 (8.7) years. In both RA groups, the assignment guess was more likely to be correct regardless of the placebo disclosure degree; 13 (45%) and 11 (35%) participants in the RA-FD and RA-PD groups were more likely to guess their group assignment as RA beyond chance (BI, 0.45 [95% CI, 0.15 to 0.76]) and 0.35 [95% CI, 0.06 to 0.63], respectively). In contrast, 11 (36%) PA-PD recipients were more likely to believe they received RA vs 9 (14%) PA-FD recipients (incorrect guess; BI, −0.36 [95% CI, −0.66 to −0.07] and −0.14 [95% CI, −0.48 to 0.20], respectively). The blinding scenario under FD of placebo information might be interpreted as possibly problematic (RA: correct guess; PA: random guess) and that of PD ideal (RA: correct guess; PA: opposite guess), resulting in a summed BI value of −0.01 ≈ 0 (Table).^5^

**Table.** Blinding Index Values for Treatment Assignment and Placebo Disclosure Status of 89 Participants

Group assignment	Guess, No. of participants (%)			BI (95% CI)^a^	Summed BI^b^	Blinding status/scenario	Possible blinding effectiveness interpretations^c^
	Acupuncture	Placebo	Do not know				
Full disclosure							
Acupuncture	13 (59)	3 (14)	6 (27)	0.45 (0.15 to 0.76)	0.31	More correct guess	Possibly problematic; there was a perceived treatment effect in the acupuncture group, whereas there was no significant effect in the placebo group (eg, placebo group participants may not have wishful thinking in the absence of a treatment effect)
Placebo	9 (41)	6 (27)	7 (32)	−0.14 (−0.48 to 0.20)		Random guess	
Partial disclosure							
Acupuncture	11 (48)	3 (13)	9 (39)	0.35 (0.06 to 0.63)	−0.01	More correct guess	Ideal; both groups tend to believe they received real acupuncture (eg, participants tend to have strong wishful thinking, or placebo as well as real acupuncture is perceived as a real treatment)
Placebo	11 (50)	3 (14)	8 (36)	−0.36 (−0.66 to −0.07)		Opposite guess	
Total	44 (49)	15 (17)	30 (34)	NA	NA	NA	NA

Abbreviations: BI, blinding index; NA, not applicable.

^a^ The BI is a chance-corrected measure of potential unblinding via a proxy for disproportionate correct guesses in a group. The BI is used to calculate the index value and then to possibly categorize blinding scenarios (using conventional, ad hoc cut points) as more correct (≥0.2), random (−0.2 < BI < 0.2), or more incorrect (≤−0.2) and another ideal blinding (−0.2 < summed BI < 0.2).

^b^ The summed BI (acupuncture group plus placebo group) measures the difference in proportions with the same guess. For values of 0, an equal proportion of participants in both groups believed they had received real acupuncture (more commonly) or placebo (less commonly), implying so-called wishful thinking (or negative thinking for placebo, which is less common). For values greater than 0, more participants in the acupuncture group believed that they had received acupuncture than those in the placebo group.

^c^ A value of 0 for either BI in both active and placebo groups or summed BI indicates the 2 most ideal blinding scenarios, implying a random guess or wishful thinking (as a metaphor for common representative scenarios), respectively.^4,5^ If both groups guessed they were receiving active treatment in equal proportions, blinding could be a success.

There were no significant between-group differences in pain and discomfort at any time point (all *P* ≥ .26). After debriefing, most participants accepted the study hypothesis, design, and overall procedures.

## Discussion

In the informed consent process, differently disclosed information about the placebo control has led to different blinding scenarios. Although research on this topic is scarce in RCT communities, how placebo information is disclosed in PILs may possibly influence participant blinding. Limitations of this study include its small sample size, the short-term trial length with an acute condition, and participants’ understanding and recollection of PILs possibly affected by other factors.

Given that PD was acceptable to most participants, further investigation is warranted to identify best practices regarding placebo disclosure to inform participants about placebo without jeopardizing their autonomy, while securing blinding and retention or adherence to ensure study rigor and validity. It would be interesting to see how these findings are translated into other medical disciplines.

## Notes

Supplement 1.
Trial Protocol

Supplement 2. **eAppendix 1.** Study Procedures **eAppendix 2.** Induction of Delayed-Onset Muscle Soreness and Outcome Measurement **eAppendix 3.** Acupuncture Protocol **eFigure.** Study Procedures

Supplement 3.
Data Sharing Statement
