# Supplementary material for: Effect of Partial vs Full Disclosure of Potential Assignment to Placebo on Participant Blinding, Perceptions of Group Assignment, and Trial Outcomes: A Randomized Clinical Trial
Source: JAMA Netw Open. 2022 Mar 24;5(3):e224050. doi: 10.1001/jamanetworkopen.2022.4050 (PMC8948530; doi:10.1001/jamanetworkopen.2022.4050)
Supplement: Supplement 1. — Trial Protocol [file jamanetwopen-e224050-s001.pdf]

Study Protocol

Statistical plan: 13-14 page

1. Summary of study protocol

**1.1 Investigator information**						
**1.1.1 Study title**	Effect of information on placebo in the informed consent on the outcome and blinding of participants in a trial					
**1.1.2 Primary investigator**	☑ **Full-time faculty** ☐ **Non-full-time faculty**					
	**Name**	**Department**	**Position**	**Phone**	**Email**	**Role**
	Hyangsook Lee	College of Korean Medicine	Professor	+82 2-961-0703	erc633@khu.ac.kr	Primary investigator
**1.2 Study information**						
**1.2.1 Study design (multiple choices allowed)**	☑ Clinical study (☐ Survey ☐ Observational study ☐ Behavior study ☑ Clinical trial ☐ Education program ☐ Sensory test)<br>☐ Human derived materials (☐ Archived samples ☐ Newly collected samples)<br>☐ Genetic research<br>☐ Personal information research<br>☐ Others ( )					
**1.2.2 Study objectives**	In randomized clinical trials, information on placebo acupuncture in informed consent documents is fully disclosed or partially disclosed to potential participants. This study aims to investigate the impact of placebo information on the study results and blinding of participants in a design of randomized trial involving healthy volunteers.					
**1.2.3 Study period**	One year from IRB approval date					
**1.2.4 Study place**	At Acupuncture and Meridian Science Research Centre in Kyung Hee University					
**1.3 Participants**						
**1.3.1 Condition and number**	Healthy 89 adults					
**1.3.2 Recruitment**	- Potential participants will be recruited via university bulletin board advertisements and via online, e.g., Every-time application and Facebook.<br>- After receiving application e-mail, volunteers will be screened for eligibility.<br>- Assessment of participant's current status through physical examination and questionnaire					
**1.3.3 Informed consent**	- At the beginning of the study, one of the two informed consent forms that differently disclosed placebo information (fully disclosed vs. partially disclosed) will be randomly given to participants. They will be informed not the true					

hypothesis but alternative hypothesis of the study, i.e., to investigate the effect of acupuncture for this delayed onset muscle soreness.
- At the end of the study, the genuine study hypothesis will be debriefed to participants that this study is not to investigate the effect of acupuncture treatment for delayed muscle pain, but to examine the impact of the placebo information in informed consent form on the study results and blinding.
- Only data of participants who provide the written informed consent after debriefing will be subject to analysis.

1.3.4 Securing safety

- Procedures of this study will be conducted following the Declaration of Helsinki (South Africa Amendment, 1996. 10).
- Research personnel who are fully aware of the adverse events and precautions will notify IRB of any serious adverse events.

1.4 Study design

1.4.1 Methods

A 2X2 factorial design randomized clinical trial will be conducted in 4 groups of healthy people.
- Two types of acupuncture: real acupuncture and placebo acupuncture
- Two types of informed consent: complete disclosure of false acupuncture information and incomplete disclosure of false acupuncture information

Acupuncture / Informed consent	Real acupuncture (RA)	Placebo acupuncture (PA)
Full disclosure of placebo information (FD)	RA-FD group	PA-FD group
Partical disclosure of placebo information (PD)	RA-PD group	PA-PD group

① Intervention 1: acupuncture
- Real acupuncture: 0.25X40mm stainless-steel needle (Dongbang, Korea)
- Placebo acupuncture: 0.25X40mm Park Sham Needle (AcuPrime, UK)

② Intervention 2: placebo information in informed consent document
- Fully disclosed information: to state the information about placebo acupuncture as it is:
"You will be randomly given experimental acupuncture or control acupuncture. Though control acupuncture mimics experimental acupuncture, it does not penetrate the skin because it is fake acupuncture with a blunt needle tip."
- Partially disclosed information: not to state the information about placebo acupuncture as it is:
"You will be randomly given experimental acupuncture or control acupuncture. Control acupuncture is a different kind of acupuncture which has been frequently used in clinical trials as a comparison to experimental acupuncture."

1.4.2 Outcome assessment

Day	Outcome assessment
1	1. Obtaining written Informed consent<br><br>2. Screening<br>- Measurements of height and weight<br>- To check the history of past/present illness, smoking/drinking, and resistance training

		- To fill out Myers credibility index checking overall validity of informed consent document<br>- To fill out Acupuncture Belief Scale (ABS) and State-Trait Anxiety Inventory-X (STAI-X)<br>- To measure pressure pain phreshold (PPT) in the non-dominant upper arm<br><br>3. Induction of delayed onset muscle soreness model<br>- Measurement of 1 Repetition Maximum (1RM) in the non-dominant arm<br>- Repetitive arm-curl exercise in the non-dominant arm using 1RM weighted dumbbell<br>- Measurement of muscle discomfort in the non-dominant arm with visual analogue scale (VAS)<br>- Measurement of PPT in the non-dominant arm<br><br>4. Acupuncture treatment<br>- RA (PC2, LI4, LI11 and LU5 of the non-dominant arm) and PA (two points on the muscle belly of biceps brachii, 1 cun proximedially from LI11, and 1 cun laterally from PC2 of the non-dominant arm) will be performed for 15 minutes once daily for three consecutive days with manipulation (by rotating 180 degrees clockwise and counter-clockwise in a frequency of 2 Hz at immediately, 3, 6, 9, and 12 minutes after needle insertion)<br><br>5. After acupuncture treatment<br>- To fill out de-qi questionnaire<br>- Measurement of muscle discomfort in the non-dominant arm with VAS<br>- Measurement of PPT in the non-dominant arm
	2	1. Before acupuncture treatment<br>- Adverse event monitoring<br>- Measurement of muscle discomfort in the non-dominant arm with VAS<br>- Measurement of PPT in the non-dominant arm<br><br>2. Acupuncture treatment<br>- RA (PC2, LI4, LI11 and LU5 of the non-dominant arm) and PA (two points on the muscle belly of biceps brachii, 1 cun proximedially from LI11, and 1 cun laterally from PC2 of the non-dominant arm) will be performed for 15 minutes once daily for three consecutive days with manipulation (by rotating 180 degrees clockwise and counter-clockwise in a frequency of 2 Hz at immediately, 3, 6, 9, and 12 minutes after needle insertion)<br><br>3. After acupuncture treatment<br>- To fill out de-qi questionnaire<br>- Measurement of muscle discomfort in the non-dominant arm with VAS<br>- Measurement of PPT in the non-dominant arm
	3	1. Before acupuncture treatment<br>- Adverse event monitoring<br>- Measurement of muscle discomfort in the non-dominant arm with

	VAS<br>- Measurement of PPT in the non-dominant arm<br><br>2. Acupuncture treatment<br>- RA (PC2, LI4, LI11 and LU5 of the non-dominant arm) and PA (two points on the muscle belly of biceps brachii, 1 cun proximedially from LI11, and 1 cun laterally from PC2 of the non-dominant arm) will be performed for 15 minutes once daily for three consecutive days with manipulation (by rotating 180 degrees clockwise and counter-clockwise in a frequency of 2 Hz at immediately, 3, 6, 9, and 12 minutes after needle insertion)<br><br>3. After acupuncture treatment<br>- To fill out de-qi questionnaire<br>- Measurement of muscle discomfort in the non-dominant arm with VAS<br>- Measurement of PPT in the non-dominant arm<br>- To fill out acupuncture credibilityredibility Test<br>- Assessment of blinding effectiveness<br><br>4. Debriefing<br>- Debriefing to the type of acupuncture and informed consent form and interviewing about the other type of informed consent form<br>- Debriefing to the genuine study hypothesis and asking to participants whether the study design is acceptable and whether they can provide informed consent about the genuine study design and analysis their data<br>- Data of participants who decided not to provide written informed consent at the end of the study will not be analyzed.
**1.4.3 Main outcomes**	Main outcomes are pain outcomes and blinding of participants<br>- Pain outcomes: PPT and VAS.<br>✓ Two way ANOVA is considered as the way of statistical plan as it can analyze the effect of two interventions (acupuncture and informed consent)<br>✓ The effect will be assessed as statistically significant when p value is under 0.05.<br><br>- Blinding of participants will be analyzed using a Blinding Index (BI, Bang, 2004).

I submit this study protocol to Institutional Review Board of Kyung Hee University.

Date: 2019/07/31

Primary investigator: Hyangsook Lee

2. Details of study protocol

2.1 Study information	
**2.1.1 Funding**	National Research Foundation (NRF) of Korea
**2.1.2 Study objectives**	In randomized clinical trials, information on placebo acupuncture in informed consent documents is fully disclosed or partially disclosed to potential participants. This study aims to investigate the impact of placebo information on the study results and blinding of participants in a design of randomized trial involving healthy volunteers.
**2.1.3 Background**	1. Information in informed consent forms<br>- Since the declaration of Helsinki has presented the ethical principles for clinical trials that potential participants should be provided with adequate information regarding study such as study objectives and procedure, most clinical studies are conducted in accordance with the declaration.<br>- Current medical studies are also conducted in accordance with the four principles of medical ethics (autonomy, non-maleficence, beneficence, and justice). (Gillon, 2003; Harris, 2003).<br>- Sufficient information in participant information leaflets or informed consent forms provided to potential participants facilitates informed consent which is assumed to serve principles of medical ethics, especially autonomy.<br>- An informed consent document is one of the most common ways to provide key information of clinical trials to potential participants. In key information is included study objectives, procedures, possible harms and benefits, and alternatives to participation.<br><br>2. Placebo information in informed consent forms of randomized clinical trials<br>- A recent study revealed that informed consent documents of qualified randomized clinical trials provide ample information on the benefits of the test intervention while the information on placebo control was lacking or inaccurate and there is a room for improvement in the current standard of informed consent documents (Bishop, 2012)<br>- Another study also presented that some of informed consent documents of acupuncture randomized clinical trials partially provided placebo information or omitted placebo information and this can affect blinding of participants and directions of trial outcomes (Cheon, 2018).<br><br>3. Previous randomized clinical trials that manipulated placebo information at the beginning of the study and debriefed true placebo information or true study hypothesis at the end of study.<br>- A recent randomized clinical trial investigated whether perception of migraineurs toward assigned intervention, I.e., a real drug or placebo, influenced the effect size of drug or placebo with hiding true study objectives at the beginning and debriefing true hypothesis. This study design facilitates (Kam-Hansen, 2014).<br>- Another randomized clinical trial provided participants with placebo and informed it is a drug that is frequently used. This study aimed to investigate the impact of framing of side effect on adverse event reporting randomized participants into differently, I. e., positively or negatively. At the end of the study, participants were debriefed to the

	true study hypothesis and requested to assess the validity of study. Participants said that the study design is acceptable (Webster 2018).<br>- To sum up, information in informed consent document may affect participants and it can be acceptable if study is well designed, e.g., at the end of the trial, participants are debriefed to true study hypothesis.<br><br>▷ In this context, this randomized clinical trial adopting delayed onset muscle soreness model in healthy participants aims to investigate the impact of placebo information disclosure in informed consent documents on blinding of participants and trial outcomes.
**2.1.4 Implications for researches**	By conducting this randomized clinical trial, we can present realistic guidelines on how to describe placebo information in the informed consent form..
**2.2 Participants**	
**2.2.1 Eligibility criteria**	■ Inclusion criteria<br>- Eighteen years of age or older<br>- Those who agree not to participate in other clinical studies during the study period<br>- Those who provide written informed consent after understanding the purpose of this study<br><br>■ Exclusion criteria<br>- Those who regularly do resistance training at least twice a week for not less than 6 months (Shmano, 2006; Graves, 1988; Yeon 2012)<br>- Those who have had surgery on bones of four limbs or have a defect in any of four limbs<br>- Pregnant or lactating women<br>- Those who with contact dermatitis and other diseases that make it difficult to be given acupuncture treatment<br>- Those who are taking corticosteroids, analgesics, muscle relaxants, and/or anticoagulants.
**2.2.2 Drop-out criteria**	Research personnel, the ethics committee, or associated institutions can consider drop-out of participants if it is necessary. Details are as follows:<br>- In case of failure to complete the first day procedure<br>- If participants do not follow instructions of research personnel<br>- When discontinuation of trial participation is optimal for a participant because he or she experiences an severe adverse event
**2.2.3 Termination of study**	Research personnel, the ethics committee, or associated institutions can decide the termination of study if it is necessary. Details are as follows:<br>- When the termination is optimal for participants because a number of severe adverse events occur.<br>- Other cases that need termination
**2.2.4 Sample size calculation**	For this is hypothesis-generating study, calculation of sample size using existing effect size is not applicable. As an exploratory study, a total of 89 people will be recruited considering the dropout rate of 10%, with the goal of recruiting 80 participants.

**2.2.5 Recruitment of participants**	- Advertisement on websites and university bulletin boards<br>- Potential participants who voluntarily send email will be screened for eligibility<br>- Demographic information of participants will be collected.<br>- Conditions of participants will be assessed by physical examination and interview with a Korean Medicine Doctor (KMD)
**2.2.6 Informed consent**	- At the beginning of the study, one of the two informed consent forms that differently disclosed placebo information (fully disclosed vs. partially disclosed) will be randomly given to participants. They will be informed not the true hypothesis but alternative hypothesis of the study, i.e., to investigate the effect of acupuncture for this delayed onset muscle soreness.<br>- At the end of the study, the genuine study hypothesis will be debriefed to participants that this study is not to investigate the effect of acupuncture treatment for delayed muscle pain, but to examine the impact of the placebo information in informed consent form on the study results and blinding.<br>- Only data of participants who provide the written informed consent after debriefing will be subject to analysis.
**2.2.7 Countermeasures for vulnerable people**	Countermeasures are not applicable for this study because vulnerable peple will not participate.
**2.2.8 Monetary incentives and possible benefits for study participation**	- Participants in this study will be paid thirty thousand won for each visit.<br>- Although benefits in symptom improvement is not expected by the study participation, knowledge or insights derived from results of this study can contribute to improvement in research methodology regarding the disclosure of placebo information in informed consent documents of randomized clinical trials.
**2.2.9 Possible harms, risks, and discomfort of study participation**	- Adverse events refer to undesired and unintended signs (e.g., abnormalities in laboratory tests), symptoms, or diseases occurring after the treatment in the study. The events do not necessarily have causal relationship with the treatment.<br><br>- Assessment of the severity (following Common Terminology Criteria for Adverse Events, CTCAE)<br>√ Grade 1 Mild; asymptomatic or mild symptoms; clinical or diagnostic observations only; intervention not indicated.<br>√ Grade 2 Moderate; minimal, local or noninvasive intervention indicated; limiting age appropriate instrumental ADL*.<br>√ Grade 3 Severe or medically significant but not immediately life-threatening; hospitalization or prolongation of hospitalization indicated; disabling; limiting self care ADL**.<br>√ Grade 4 Life-threatening consequences; urgent intervention indicated.<br>√ Grade 5 Death related to adverse event.<br><br>- Assessment of the causality (following WHO-UMC Causality Categories)<br>√ Certain<br>• Event or laboratory test abnormality, with plausible time relationship to drug intake<br>• Cannot be explained by disease or other drugs

	• Response to withdrawal plausible (pharmacologically, pathologically)<br>• Event definitive pharmacologically or phenomenologically (i.e. an objective and specific medical disorder or a recognised pharmacological phenomenon)<br>• Rechallenge satisfactory, if necessary<br>√ Probable/Likely<br>• Event or laboratory test abnormality, with reasonable time relationship to drug intake<br>• Unlikely to be attributed to disease or other drugs<br>• Response to withdrawal clinically reasonable<br>• Rechallenge not required<br>√ Possible<br>• Event or laboratory test abnormality, with reasonable time relationship to drug intake<br>• Could also be explained by disease or other drugs<br>• Information on drug withdrawal may be lacking or unclear<br>√ Unlikely<br>• Event or laboratory test abnormality, with a time to drug intake that makes a relationship improbable (but not impossible)<br>• Disease or other drugs provide plausible explanations<br>√ Conditional/Unclassified<br>• Event or laboratory test abnormality<br>• More data for proper assessment needed, or<br>• Additional data under examination<br>√ Unassessable/Unclassifiable<br>• Report suggesting an adverse reaction<br>• Cannot be judged because information is insufficient or contradictory<br>• Data cannot be supplemented or verified<br><br>- Reporting of adverse events: adverse events will be analyzed by the investigator who will be in charge of examining the reaction suspected of the correlation with the treatment, abnormalities in the laboratory test results, and the frequency of serious adverse events occurrence. The collected safety data will be appropriately documented. All serious adverse events will be descriptively written. Adverse events will be collected through the patient's symptom report and investigator's observation and monitoring.
**2.2.10 Possible disadvantages regarding voluntary discontinuation of participation**	- Participants will not be obligated to participate in this study, and even if they agree to participate, they can decide voluntary discontinuation of participation at any time.<br>- No disadvantages will be imposed to the discontinuation
**2.3.1 Study period**	One year from IRB approval date
**2.3.2 Study place**	At Acupuncture and Meridian Science Research Centre in Kyung Hee University
**2.3.3 Study design**	A 2X2 factorial design randomized clinical trial will be conducted in 4 groups of healthy people.<br>- Two types of acupuncture: real acupuncture and placebo acupuncture

- Two types of informed consent: complete disclosure of false acupuncture information and incomplete disclosure of false acupuncture information

Informed consent \ Acupuncture	Real acupuncture (RA)	Placebo acupuncture (PA)
Full disclosure of placebo information (FD)	RA-FD group	PA-FD group
Partical disclosure of placebo information (PD)	RA-PD group	PA-PD group

① Intervention 1: acupuncture
- Real acupuncture: 0.25X40mm stainless-steel needle (Dongbang, Korea)
- Placebo acupuncture: 0.25X40mm Park Sham Needle (AcuPrime, UK)

② Intervention 2: placebo information in informed consent document
- Fully disclosed information: to state the information about placebo acupuncture as it is:
"You will be randomly given experimental acupuncture or control acupuncture. Though control acupuncture mimics experimental acupuncture, it does not penetrate the skin because it is fake acupuncture with a blunt needle tip."
- Partially disclosed information: not to state the information about placebo acupuncture as it is:
"You will be randomly given experimental acupuncture or control acupuncture. Control acupuncture is a different kind of acupuncture which has been frequently used in clinical trials as a comparison to experimental acupuncture."

2.3.4 Study schedule

Period after approval	Details
One month	Recruitment of participants
Three to five months	Conduct of randomized clinical trials
Six to twelve months	Data analysis and writing manuscript
One year	Submission of trial result report to the institutional review board
Three years	Discarding data

Day	Outcome assessment
1	1. Retrieval of written Informed consent<br><br>2. Screening<br>- Measurement of height and weight<br>- To check history of past/present illness, smoking/drinking, and resistance training<br>- To fill out Myers credibility index checking overall validity of informed consent document<br>- To fill out ABS and STAI-X<br>- To measure PPT in the non-dominant upper arm

		3. Induction of delayed onset muscle soreness model<br>- Measurement of 1RM in the non-dominant arm<br>- Repetitive arm-curl exercise in the non-dominant arm using 1RM weighted dumbbell<br>- Measurement of muscle discomfort in the non-dominant arm with VAS<br>- Measurement of PPT in the non-dominant arm<br><br>4. Acupuncture treatment<br>- RA (PC2, LI4, LI11 and LU5 of the non-dominant arm) and PA (two points on the muscle belly of biceps brachii, 1 cun proximedially from LI11, and 1 cun laterally from PC2 of the non-dominant arm) will be performed for 15 minutes once daily for three consecutive days with manipulation (by rotating 180 degrees clockwise and counter-clockwise in a frequency of 2 Hz at immediately, 3, 6, 9, and 12 minutes after needle insertion)<br><br>5. After acupuncture treatment<br>- To fill out de-qi questionnaire<br>- Measurement of muscle discomfort in the non-dominant arm with VAS<br>- Measurement of PPT in the non-dominant arm
	2	1. Before acupuncture treatment<br>- Adverse event monitoring<br>- Measurement of muscle discomfort in the non-dominant arm with VAS<br>- Measurement of PPT in the non-dominant arm<br><br>2. Acupuncture treatment<br>- RA (PC2, LI4, LI11 and LU5 of the non-dominant arm) and PA (two points on the muscle belly of biceps brachii, 1 cun proximedially from LI11, and 1 cun laterally from PC2 of the non-dominant arm) will be performed for 15 minutes once daily for three consecutive days with manipulation (by rotating 180 degrees clockwise and counter-clockwise in a frequency of 2 Hz at immediately, 3, 6, 9, and 12 minutes after needle insertion)<br><br>3. After acupuncture treatment<br>- To fill out de-qi questionnaire<br>- Measurement of muscle discomfort in the non-dominant arm with VAS<br>- Measurement of PPT in the non-dominant arm
	3	1. Before acupuncture treatment<br>- Adverse event monitoring<br>- Measurement of muscle discomfort in the non-dominant arm with VAS<br>- Measurement of PPT in the non-dominant arm<br><br>2. Acupuncture treatment<br>- RA (PC2, LI4, LI11 and LU5 of the non-dominant arm) and PA (two points on the muscle belly of biceps brachii, 1 cun proximedially from LI11, and 1 cun laterally from PC2 of the non-dominant arm) will be performed for 15 minutes once daily

	for three consecutive days with manipulation (by rotating 180 degrees clockwise and counter-clockwise in a frequency of 2 Hz at immediately, 3, 6, 9, and 12 minutes after needle insertion)<br><br>3. After acupuncture treatment<br>- To fill out de-qi questionnaire<br>- Measurement of muscle discomfort in the non-dominant arm with VAS<br>- Measurement of PPT in the non-dominant arm<br>- To fill out acupuncture credibility Test<br>- Assessment of blinding effectiveness<br><br>4. Debriefing<br>- Debriefing to the type of acupuncture and informed consent form and interviewing about the other type of informed consent form<br>- Debriefing to the genuine study hypothesis and asking to participants whether the study design is acceptable and whether they can provide informed consent about the genuine study design and analysis their data<br>- Data of participants who decided not to provide written informed consent at the end of the study will not be analyzed.
**2.3.5 Random sequence generation, allocation concealment, and blinidng**	■ Randomization<br>- Random sequence generation<br>✓ The random sequence will be generated using Microsoft's Excel 2016 version.<br>- Allocation concealment<br>✓ Randomization of participants is carried out by a separate researcher who will not be related with other study procedures, and the random sequence will placed in an opaque and sealed envelope so that identification of the sequence is not available.<br><br>■ Blinding<br>- Blinding of participants<br>✓ Participants cannot discriminate two acupuncture, I.e., RA and PA, because appearance of PA is similar to that of RA (Park, 1999).<br>- Blinding of practitioner and outcome assessment<br>✓ Blinding of practitioner is not available considering the nature of intervention (acupuncture) and blinding of outcome assessment (PPT and VAS) will be achieved in that a separate researcher (not the practitioner) will measure the outcomes
**2.3.6 Intervention protocol**	(see table below)

Item	Details
1. Acupuncture rationale	1a) Traditional Korean Medicine style acupuncture<br>1b) Based on theories of Traditional Korean Medicine<br>1c) Extent to which treatment was varied: not

<table>
<tr><td></td><td>applicable</td></tr>
<tr><td>2. Details of needling</td><td>2a) Number of needle insertions per subject per session: four needles
2b) PC2, LU5, LI11, and LI4 in the non-dominant arm
2c) Depth of insertion: 10-20mm
2e) Needle stimulation: manual
2f) Needle retention time: 15 minutes
2g) Needle type: 0.25X40mm stainless-steel needle (Dongbang, Korea)</td></tr>
<tr><td>3. Treatment regimen</td><td>3a) Number of treatment sessions: three sessions
3b) Frequency and duration of treatment sessions: once daily for three consecutive days</td></tr>
<tr><td>4. Other components of treatment</td><td>4a) Details of other interventions administered to the acupuncture group: not applicable
4b) Setting and context of treatment, including instructions to practitioners, and information and explanations to patients: healthy participants who are induced delayed onset muscle soreness will be give acupuncture in research setting</td></tr>
<tr><td>5. Practitioner background</td><td>Qualified Korean Medicine Doctor</td></tr>
<tr><td>6. Control or comparator interventions</td><td>Control: Park Sham needle
<table>
<tr><th>Item</th><th>Details</th></tr>
<tr><td>1. Acupuncture rationale</td><td>1a) Traditional Korean Medicine style acupuncture
1b) Based on theories of Traditional Korean Medicine
1c) Extent to which treatment was varied: not applicable</td></tr>
<tr><td>2. Details of needling</td><td>2a) Number of needle insertions per subject per session: four needles
2b) two points on muscle belly of biceps brachii, 1 cun proximedially from LI11, and 1 cun laterally from PC2 in the non-dominant arm
2c) Depth of insertion: 0mm
2e) Needle stimulation: manual
2f) Needle retention time: 15 minutes
2g) 0.25X40mm Park Sham Needle (AcuPrime, UK)</td></tr>
<tr><td>3. Treatment regimen</td><td>3a) Number of treatment sessions: three sessions
3b) Frequency and duration of treatment sessions: once daily for three consecutive days</td></tr>
</table></td></tr>
</table>

**2.3.9 Statistical plan**	Main outcomes are pain outcomes and blinding of participants<br>- Pain outcomes: PPT and VAS.<br>✓ Two way ANOVA is considered as the way of statistical plan as it can analyze effect of two interventions (acupuncture and informed consent)<br>✓ The effect will be assessed as statistically significant when p value is under 0.05.<br><br>- Blinding of participants will be analyzed using a Blinding Index (BI, Bang, 2004).<br><br>- Scores of Myers credibility index, ABS, STAI-X, acupuncture credibility test, and de-qi questionnaire will be analyzed using two-way ANOVA with independent variables (type of acupuncture and placebo information disclosure). A p value less than 0.05 will be considered as statistically significant.<br><br>- Adverse events will be reported with details and causal relationship with acupuncture will be also assessed. Whether frequency of adverse events that has causal relationship with acupuncture differ between acupuncture types will be analyzed using chi-squared test or Fisher's exact test. A p value less than 0.05 will be considered as statistically significant.<br><br>- Demographic data will reported descriptively, i.e., continuous variables will be described with mean ± standard deviation and categorical variables will be shown as frequency (%).
**2.3.10 Safety securing**	- Monitoring adverse events<br>- Interviewing with participants at day 2 and day 3 visit<br>- Any adverse event will be described with details and numbers of adverse events have causal relationship or do not have one will be also described.<br>- Adverse events that are frequently reported following acupuncture: bruising, local pain, and minor bleeding (MacPherson, 2001)<br><br>- Adverse events refer to undesired and unintended signs (e.g., abnormalities in laboratory tests), symptoms, or diseases occurring after the treatment in the study. The events do not necessarily have a causal relationship with the treatment.<br><br>- Assessment of the severity (following Common Terminology Criteria for Adverse Events, CTCAE)<br>√ Grade 1 Mild; asymptomatic or mild symptoms; clinical or diagnostic observations only; intervention not indicated.<br>√ Grade 2 Moderate; minimal, local or noninvasive intervention indicated; limiting age appropriate instrumental ADL*.<br>√ Grade 3 Severe or medically significant but not immediately life-threatening; hospitalization or prolongation of hospitalization indicated; disabling; limiting self care ADL**.<br>√ Grade 4 Life-threatening consequences; urgent intervention indicated.<br>√ Grade 5 Death related to adverse event.<br><br>- Assessment of the causality (following WHO-UMC Causality Categories)<br>√ Certain

	• Event or laboratory test abnormality, with plausible time relationship to drug intake<br>• Cannot be explained by disease or other drugs<br>• Response to withdrawal plausible (pharmacologically, pathologically)<br>• Event definitive pharmacologically or phenomenologically (i.e. an objective and specific medical disorder or a recognised pharmacological phenomenon)<br>• Rechallenge satisfactory, if necessary<br>√ Probable/Likely<br>• Event or laboratory test abnormality, with reasonable time relationship to drug intake<br>• Unlikely to be attributed to disease or other drugs<br>• Response to withdrawal clinically reasonable<br>• Rechallenge not required<br>√ Possible<br>• Event or laboratory test abnormality, with reasonable time relationship to drug intake<br>• Could also be explained by disease or other drugs<br>• Information on drug withdrawal may be lacking or unclear<br>√ Unlikely<br>• Event or laboratory test abnormality, with a time to drug intake that makes a relationship improbable (but not impossible)<br>• Disease or other drugs provide plausible explanations<br>√ Conditional/Unclassified<br>• Event or laboratory test abnormality<br>• More data for proper assessment needed, or<br>• Additional data under examination<br>√ Unassessable/Unclassifiable<br>• Report suggesting an adverse reaction<br>• Cannot be judged because information is insufficient or contradictory<br>• Data cannot be supplemented or verified

2.5 References

- ✓ Kim Y. (2006). Development, Validity and Reliability Testing of a Deqi Sensation Questionnaire by In-depth Interview. (Master's thesis). Kyung Hee University.
- ✓ Kim J. (1978). Association of stait-anxiety with sociability. (Master's thesis). Korea University.
- ✓ Yeon C, Chung S. (2012). Comparison of Efficacy between Acupuncture Treatment at Muscle Belly and Acupuncture Treatment at Myotendinal Junction on Delayed-Onset Muscle Soreness. Journal of Oriental Rehabilitation Medicine, 22(2), 219-228.
- ✓ Bang, H., Ni, L., & Davis, C. E. (2004). Assessment of blinding in clinical trials. Controlled Clinical Trials, 25(2), 143-156.
- ✓ Bishop, F. L., Adams, A. E., Kaptchuk, T. J., & Lewith, G. T. (2012). Informed consent and placebo effects: a content analysis of information leaflets to identify what clinical trial participants are told about placebos. PLoS One, 7(6), e39661.
- ✓ Cheon, S., Park, H. J., Chae, Y., & Lee, H. (2018). Does different information disclosure on placebo control affect blinding and trial outcomes? A case study of participant information leaflets of randomized placebo-controlled trials of acupuncture. BMC Medical Research Methodology, 18(1), 13.
- ✓ Corder, K. E., Newsham, K. R., McDaniel, J. L., Ezekiel, U. R., & Weiss, E. P. (2016). Effects of short-term docosahexaenoic acid supplementation on markers of inflammation after eccentric strength exercise in women. Journal of Sports Science & Medicine, 15(1), 176.

- ✓ Dennehy EB., Webb A., Suppes T. (2002). Assessment of beliefs in the effectiveness of acupuncture for treatment of psychiatric symptoms. Journal of Complementary Medicine, 8(4), 421-425.
- ✓ Fleckenstein J., Niederer D., Auerbach K., Bernhörster M., Hübscher M., Vogt L., et al. (2016). No effect of acupuncture in the relief of delayed-onset muscle soreness: results of a randomized controlled trial. Clinical Journal of Sport Medicine, 26(6), 471-477.
- ✓ Fleckenstein J., Simon P., König M., Vogt L., Banzer W. (2017). The pain threshold of high-threshold mechanosensitive receptors subsequent to maximal eccentric exercise is a potential marker in the prediction of DOMS associated impairment. PLoS One, 12(10), e0185463.
- ✓ Gillon, R. (2003). Ethics needs principles—four can encompass the rest—and respect for autonomy should be "first among equals". Journal of Medical Ethics, 29(5), 307-312.
- ✓ Graves JE., pollock ML., Leggett SH., Braith RW., Carpenter DM., Bishop LE. (1988), Effect of reduced training frequency on muscular strength. International Journal of Sports Medicine, 9(5), 316-319.
- ✓ Harris, J. (2003). In praise of unprincipled ethics. Journal of Medical Ethics, 29(5), 303-306.
- ✓ Hübscher, M., Vogt, L., Bernhörster, M., Rosenhagen, A., & Banzer, W. (2008). Effects of acupuncture on symptoms and muscle function in delayed-onset muscle soreness. The Journal of Alternative and Complementary Medicine, 14(8), 1011-1016.
- ✓ Hutchins, M., & Gearhart Jr, R. (2010). Accuracy of 1-RM Prediction Equations for the Bench Press and Biceps Curl. Journal of Exercise Physiology Online, 13(3).
- ✓ Kam-Hansen S., Jakubowski M., Kelley JM., Kirsch I., Hoaglin DC., Kaptchuk TJ., et al. (2014). Altered placebo and drug labeling changes the outcome of episodic migraine attacks Science Translational Medicine., 6(218), 218ra5.
- ✓ Lin, M. J., Nosaka, K., Ho, C. C., Chen, H. L., Tseng, K. W., Ratel, S., & Chen, T. C. C. (2018). Influence of maturation status on eccentric exercise-induced muscle damage and the repeated bout effect in females. Frontiers in Physiology, 8, 1118.
- ✓ MacPherson H., Thomas K., Walters S., Fitter M. (2001). The York acupuncture safety study: prospective survey of 34,000 treatments by traditional acupuncturists. BMJ, 323(7311), 486-487.
- ✓ Myers MG., Cairns JA., Singer J. (1987). The consent form as a possible cause of side effects. Clinical Pharmacology and Therapeutics, 42(3), 250-253.
- ✓ Park, J., White, A., Lee, H., & Ernst, E. (1999). Development of a new sham needle. Acupuncture in Medicine, 17(2), 110-112.
- ✓ Shimano T., Kraemer WJ., Spiering BA., Volek JS., Hatfield DL., Silvestre R., et al. (2006). Relationship between the number of repetitions and selected percentages of one repetition maximum in free weight exercises in trained and untrained men. Journal of Strength and Conditioning Research, 20(4), 819-823.
- ✓ Spielberger CD., Gorsuch RL., Lushene RE. (1970). Manual for the state-trait anxiety inventory. Palo alto. California: Consulting Psychologists Press.
- ✓ Webster, R. K., Weinman, J., & Rubin, G. J. (2018). Positively framed risk information in patient information leaflets reduces side effect reporting: a double-blind randomized controlled trial. Annals of Behavioral Medicine, 52(11), 920-929.
