# Supplementary material for: Effect of Partial vs Full Disclosure of Potential Assignment to Placebo on Participant Blinding, Perceptions of Group Assignment, and Trial Outcomes: A Randomized Clinical Trial
Source: JAMA Netw Open. 2022 Mar 24;5(3):e224050. doi: 10.1001/jamanetworkopen.2022.4050 (PMC8948530; doi:10.1001/jamanetworkopen.2022.4050)
Supplement: Supplement 2. — eAppendix 1. Study Procedures eAppendix 2. Induction of Delayed-Onset Muscle Soreness and Outcome Measurement eAppendix 3. Acupuncture Protocol eFigure. Study Procedures [file jamanetwopen-e224050-s002.pdf]

Supplementary Online Content

Won J, Bang H, Lee H. Effect of partial vs full disclosure of potential assignment to placebo on participant blinding, perceptions of group assignment, and trial outcomes: a randomized clinical trial. *JAMA Netw Open*. 2022;5(3):e224050. doi:10.1001/jamanetworkopen.2022.4050

eAppendix 1. Study Procedures

eAppendix 2. Induction of Delayed-Onset Muscle Soreness and Outcome Measurement

eAppendix 3. Acupuncture Protocol

eFigure. Study Procedures

This supplementary material has been provided by the authors to give readers additional information about their work.

© 2022 Won J et al. *JAMA Network Open*.

eAppendix 1. Study Procedures

Participants
This study was conducted in an academic research center at Kyung Hee University, Seoul, Korea, between October 2019 and December 2019 (NCT04182295). Advertisements via job websites and university bulletin boards informed readers of the study aim, to investigate effects of acupuncture on delayed onset muscle soreness (DOMS) omitting the true hypothesis, of the impact of placebo information disclosure in PILs on blinding and outcomes. Healthy adult volunteers were recruited and exclusion criteria included: regular resistance training at least twice weekly for more than 6 months before participation; fracture of any of 4 limbs; pregnant or breastfeeding; oral analgesic within 24 hours of participation; and any disease or condition that could compromise the safety of acupuncture treatment.

Randomization
Random numbers were generated using Microsoft Excel and concealed in sequentially numbered sealed opaque envelopes by a study staff who was only involved in randomization and assessment of pressure pain threshold (PPT) and visual analogue scale (VAS) outcomes. Volunteers meeting the inclusion criteria were randomized to one of four groups: real acupuncture (RA) and FD (RA-FD); RA-PD; placebo acupuncture (PA) and FD (PA-FD); or PA-PD. In FD, the patient information leaflet (PIL) reads, "You will be randomly given experimental acupuncture or control acupuncture. Though control acupuncture mimics experimental acupuncture, it does not penetrate the skin because it is fake acupuncture with a blunt needle tip." PD stated, "You will be randomly given experimental acupuncture or control acupuncture. Control acupuncture is a different kind of acupuncture which has been frequently used in clinical trials as a comparison to experimental acupuncture."

Statistical analysis
All statistical analyses were performed with SPSS (IBM Corp., Version 25.0, Armonk, NY, USA). Demographic data and AEs were reported descriptively, i.e., continuous variables were described with mean ± standard deviation or 95% CI and categorical variables were shown as frequency (%). After confirming assumptions of normality, variance equality, and sphericity, PPT and VAS were subjected to 3X2X2 mixed design analysis of variance (ANOVA) at three timepoints (immediately, 24 hours, and 48 hours after DOMS induction) as a within-subject factor and by type of acupuncture (RA or PA) and placebo information disclosure (FD or PD) as between-subject factors. A p-value < 0.05 was considered statistically significant.

© 2022 Won J et al. *JAMA Network Open*.

eAppendix 2. Induction of Delayed-Onset Muscle Soreness and Outcome Measurement

Induction of delayed onset muscle soreness

To experimentally induce delayed onset muscle soreness in the non-dominant biceps brachii, arm curl exercise i.e., 5 repetitions at 40% of perceived 1 repetition maximum (1 RM: the maximum amount of weight that a participant possibly lifts for 1 repetition), was performed to warm up and train participants using an adjustable 45-degree-curl bench. The eccentric exercise protocol was as follows: from fully flexed to fully extended position in 5 seconds with a uniform degree and then back to the bent position in 1 second. After 1-minute rest period, participants performed additional warm up set of 5 repetitions at 60% of perceived 1RM. Additional weight is applied in 0.5Kg increment from 6.65KG for male and 4.15Kg for female until one's 1RM was achieved. Three-minute-rest is given between attempts and number of attempts consisted of an average of 3 or 4 with a maximum of 6 attempts. After 1RM was determined, participants were instructed to lower the 1RM weighted dumbbell from fully flexed position to fully extended position for themselves. A research personnel then helped participants to lift the dumbbell holding the edge of handle. By being verbally encouraged to repeat arm curls, participants continued the exercise until they were exhausted. Exercise protocol was adjusted to fit our study.

Baseline PPT and VAS

Pressure pain threshold (PPT) was measured in a supine position on a bed using a handheld electronic pressure algometer (JTech Medical, Salt City, UT, USA) with 1cm diameter hard rubber probe that was calibrated in Newton. PPT was assessed at 5 equidistant points which were perpendicular to the biceps brachii belly of non-dominant arm on an imaginary line between the coracoid process and radial tuberosity. Applied pressure was increased at a rate of approximately 5N/cm^2 per second until a participant reported it feels painful. To familiarize participants with PPT measuring procedure, several rehearsals were performed in their lower arm before the actual measurement. Among 5 points, more proximal points were assessed earlier with an interval of 10 seconds between respective measurements. Total of 3 rounds were performed in the same manner with 2-minute interval between rounds. The baseline value was measured before model induction. Protocol of PPT measurement was modified from previous studies.

Since we included only healthy participants without any musculoskeletal diseases, participants did not report any discomfort in their non-dominant arm. Thus, the initial muscle discomfort measured with visual analogue scale (VAS, with 0 indicating no muscle discomfort at movement of several flexions and extensions and 100 indicating the worst imaginable discomfort at movement), the baseline VAS at movement was considered 0.

Questionnaires

Participants were asked to complete questionnaires such as acupuncture credibility test, Acupuncture belief scale (ABS), Myers credibility test, and state-trait anxiety inventory (STAI)-X.

Acupuncture credibility test, a 4 item self-report on a 6-point Likert scale, was used to evaluate credibility of acupuncture treatment. Higher score of the test correlates with more credibility to the treatment. Items included in acupuncture credibility test are as follows:

1. How confident do you feel that acupuncture can relieve your pain?
2. How confident would you be in recommending acupuncture to a friend who suffered from pain?
3. How logical does acupuncture seem to you?
4. How successful do you think acupuncture would be in alleviating other symptoms?

ABS, a 36-item self-report on a 5-point Likert scale enables participants to score acupuncture experiences and beliefs. The score ranges from 36 to 180 and higher score indicates more positive attitude toward acupuncture. Myers credibility index was adopted to measure the credibility of the information in a PIL by participants' rating PILs on trust, accuracy, fairness, bias, and disclosure using a 5-point Likert scale. To assess anxiety of participants, they were asked to fill in State-trait anxiety inventory-X (STAI-X). Each of the inventory consists of 20 questions on a 4-point Likert scale with higher score correlating with greater anxiety.

© 2022 Won J et al. *JAMA Network Open*.

eAppendix 3. Acupuncture Protocol

Real acupuncture
A sterilised stainless-steel needle (diameter 0.25mm X length 40mm, Dongbang Acupuncture Inc., Boryeong, Chungcheongnam-do, Korea) was given for 15 minutes at 4 acupoints including PC2, LI4, LI11 and LU5 of non-dominant arm with a depth of 10 - 20 mm once daily for 3 days (immediately after induction of delated onset muscle soreness and additional 2 consecutive days) by a qualified Korean Medicine doctor. Real acupuncture was manipulated by rotating 180 degrees clockwise and counter-clockwise in a frequency of 2 Hz at immediately, 3, 6, 9, and 12 minutes after needle insertion.

Placebo acupuncture
A Park Sham Needle (Meridus Medical Ltd & DongBang Acuprime Ltd., Exeter, UK), non-penetrating placebo acupuncture was given at 4 points including two points on muscle belly of biceps brachii, 1 cun proximedially from LI11, and 1 cun laterally from PC2 of ipsilateral arm for 15 minutes once daily for 3 days (immediately after induction of delated onset muscle soreness and additional 2 consecutive days) by the same qualified Korean Medicine doctor who gave real acupuncture treatment. Placebo acupuncture was manipulated by the same manner of real acupuncture manipulation.

© 2022 Won J et al. *JAMA Network Open*.

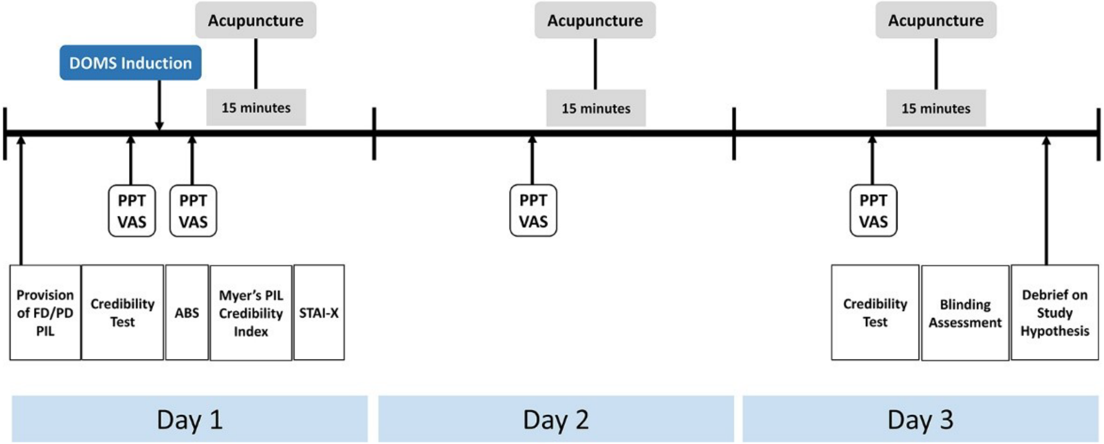

eFigure. Study Procedures

ABS, acupuncture belief scale; DOMS, delayed onset muscle soreness; FD, full disclosure; PD, partial disclosure; PIL, participant information leaflet; PPT, pressure pain threshold; STAI-X, state-trait anxiety inventory; VAS, visual analogue scale.

© 2022 Won J et al. *JAMA Network Open*.
