# Supplementary material for: Effect of Partial vs Full Disclosure of Potential Assignment to Placebo on Participant Blinding, Perceptions of Group Assignment, and Trial Outcomes: A Randomized Clinical Trial
Source: JAMA Netw Open. 2022 Mar 24;5(3):e224050. doi: 10.1001/jamanetworkopen.2022.4050 (PMC8948530; doi:10.1001/jamanetworkopen.2022.4050)
Supplement: Supplement 3. — Data Sharing Statement [file jamanetwopen-e224050-s003.pdf]

Data Sharing Statement

Won. Effect of Partial vs Full Disclosure of Potential Assignment to Placebo on Participant Blinding, Perceptions of Group Assignment, and Trial Outcomes. *JAMA Netw Open*. Published March 24, 2022. doi:10.1001/jamanetworkopen.2022.4050

Data
Data available: No

Additional Information
Explanation for why data not available: The study data are available from the corresponding author on reasonable request.
